# Efficacy of antibiotic therapy for SAPHO syndrome is lost after its discontinuation: an interventional study

**DOI:** 10.1186/ar2812

**Published:** 2009-09-21

**Authors:** Gunter Assmann, Olaf Kueck, Timm Kirchhoff, Herbert Rosenthal, Jan Voswinkel, Michael Pfreundschuh, Henning Zeidler, Annette D Wagner

**Affiliations:** 1 Department of Rheumatology, University Saarland Medical School Kirrbergerstrasse 1, D66421 Homburg, Germany; 2 Department of Anaesthesiology, Hospital of Bremerhaven, Wiener Strasse 10, D-27568 Bremerhaven, Germany; 3 Department of Radiology, University Medical School Hannover, Carl-Neuberg-Strasse 1, D-30625 Hannover, Germany; 4 Institution of Rheumatologikum Hannover, Carl-Neuberg-Strasse 1, D-30625 Hannover, Germany; 5 Department of Nephrology, University Medical School Hannover, Carl-Neuberg-Strasse 1, D-30625 Hannover, Germany

## Abstract

**Introduction:**

The acronym SAPHO was introduced in 1987 to unify the various descriptions of a seronegative arthritis associated with skin manifestations and to show synovitis, acne, pustulosis, hyperostosis, and osteitis with and without sterile multifocal osteomyelitis. The etiology of SAPHO syndrome is unknown, but an association with infection by semipathogenic bacteria like *Propionibacterium acnes *has been suggested. We conducted an interventional study of SAPHO patients receiving antibiotics.

**Methods:**

Thirty-seven patients met the clinical criteria of SAPHO syndrome, 21 of them underwent a needle biopsy of the osteitis lesion, and 14 of them showed positive bacteriological cultures for *P. acnes*. Thirty patients (14 bacteriological positive and 16 without biopsy) were treated with antibiotics for 16 weeks. The activity of skin disease and osteitis were assessed by a physician using a scoring model (from 0 to 6). In addition, patients completed a Health Assessment Score (HAS, from 0 to 6). The erythrocyte sedimentation rate was determined and a MRI (of the osteitis lesion, radiologic activity score from 0 to 2) was performed in week 1 (W1), week 16 (W16), and week 28 (W28, 12 weeks after antibiotics).

**Results:**

Twenty-seven patients continued the medication (azithromycin, n = 25, 500 mg twice a week; clindamycin, n = 1, 300 mg daily; or doxycycline, n = 1, 100 mg daily) for 16 weeks. After W16 the scores for MRI (1.5 to 1.1, *P *= 0.01), skin activity (3.2 to 1.2, *P *= 0.01), osteitis activity (4.0 to 2.1, *P *= 0.02), and HAS (3.3 to 2.1, *P *= 0.01) decreased significantly. However, this was followed by increasing values for MRI scores (1.2 to 1.4, *P *= 0.08), skin activity (1.2 to 1.7, *P *= 0.11), osteitis activity (1.9 to 2.7, *P *= 0.01), and HAS (2.2 to 3.3, *P *= 0.02) from W16 to W28. The comparison of the scores in W1 and W28 in these 12 patients showed no significant differences.

**Conclusions:**

For the period of application, the antibiotic therapy seems to have controlled the disease. After antibiotic discontinuation, however, disease relapse was observed. SAPHO syndrome thus groups with other chronic inflammatory arthropathies with a need for permanent therapy.

## Introduction

In 1987 Chamot and colleagues coined the acronym SAPHO for synovitis, acne, pustulosis, hyperostosis, and osteitis, which replaced various previously suggested descriptions of osteoarticular disease associated with skin manifestations [1,2]. The clinical feature of chronic recurrent multiple osteomyelitis (CRMO) with its typical presentation in the pediatric population justifies the inclusion of CRMO into the same nosologic group as the SAPHO syndrome according to several authors [3,4].

The etiology of these diseases is still unknown. An association with infection by semipathogenic bacteria such as *Propionibacterium acnes *has been suggested, but the role of these bacteria is discussed controversially [5,6]. Furthermore, a part of coagulase-negative *Staphyloccocus aureus *as well as *Haemophilus parainfluenzae *and *Actinomyces *were reported to be associated with SAPHO syndrome [7,8]. Family-based observations and investigations of genetic variations gave rise to the hypothesis that genetic factors contribute to the development and course of the disease [9,10]. Moreover, SAPHO syndrome shows a clear overlap with several inflammatory rheumatic diseases such as ankylosing spondylitis, psoriatic arthritis, enteropathic arthritis, reactive arthritis, and undifferentiated spondyloarthritis. In 13 to 52% of SAPHO cases, radiologic findings show sacroiliitis - as in typical ankylosing spondylitis [11]. The genetic marker HLA-B27, however, is not clearly associated with SAPHO syndrome [12,13]. Clinical symptoms of psoriatic arthritis are comparable with the features of SAPHO syndrome. In some cases, psoriasis vulgaris has developed after initial typical skin changes in patients with SAPHO syndrome. The skin manifestation of acne vulgaris is not typical of psoriatic arthritis, however, and the psoriatic-typical nail dystrophy has not been reported in SAPHO patients.

Although the classification of SAPHO syndrome exists as a distinct disease entity, the overlap and similarities with other rheumatic diseases formed the basis for trials investigating antirheumatic drugs that are the accepted standard for the treatment of psoriatic arthritis and other spondyloarthritides. Studies have been published with small numbers of patients treated with nonsteroidal anti-inflammatory drugs [14], steroids [15,16] and immunosuppressive agents that showed only partial efficacy. Investigations of methotrexate and azathioprine yielded no convincing results [17,18]. Several reports presenting promising results obtained with bisphosphonates [19-21] or biologicals like TNFα-blockers [22,23], however, have recently been published. With regard to the possible link to an infectious etiology of SAPHO syndrome, several studies with small numbers of patients treated with antibiotics reported contradictory results [24,25]. According to these studies, the antibiotic agent of azithromycin was suggested as the most promising agent for treatment of patients with SAPHO syndrome. We have therefore conducted a prospective interventional study to evaluate the efficacy of antibiotics in patients with SAPHO syndrome.

## Materials and methods

### Study design

We conducted a prospective, interventional study in patients treated at the Rheumatology Departments of the Hannover Medical School and the Saarland University Medical School, Germany. The responsible ethics committee of Hannover Medical School, Germany approved the study. The study was conducted in accordance with the Declaration of Helsinki, and each participant gave written informed consent.

### Patients

From October 1998 until February 2007 we screened 37 patients with SAPHO syndrome. All patients fulfilled the criteria of SAPHO syndrome - that is, osteitis of any location with inflammatory extra-osteoarticular manifestations of palmoplantar pustulosis, psoriasis vulgaris, or acne fulminans, with or without arthritis and/or CRMO - as defined by Chamot and colleagues [2] and by Kahn and colleagues [26].

Of 25 patients with dermatological manifestations, one patient presented with psoriasis vulgaris, 21 patients with pustulosis palmoplantaris, one patient with acne conglobata, and two patients with acne papulopustulosa (Table 1). Patients with axial disease and spondylitis were excluded in order to avoid the inclusion of patients with ankylosing spondylitis (Bechterew's disease). Twenty-one patients underwent a computed tomography-guided needle biopsy of the osteitis lesion for histopathological and bacteriological investigation as described previously [27]. Inclusion criteria were age > 18 years and clinical activity of SAPHO syndrome. Exclusion criteria were antibiotic treatment in the previous 12 weeks, women who were pregnant or breastfeeding, patients on medication with corticosteroids in a higher dosage than 10 mg prednisolone or equivalent steroids per day, psychiatric disorders that might compromise compliance with therapy, and contraindications for administration of azithromycin, doxycycline, and clindamycin, including a history of allergy to any antibiotics.

**Table 1:** Clinical characteristics of SAPHO patients on antibiotics

Clinical parameter	SAPHO patients (n = 30)
Age (years)	
Mean ± standard error	51.6 ± 3.3
Median (range)	51 (20 to 72)
Sex (female:male)	16/14 (53/47)
Disease duration (years)	
Mean ± standard error	9.4 ± 2.5
Median (range)	11 (1 to 27)
HLA B27-positive	2 (7)
Chronic recurrent multifocal osteomyelitis	4 (14)
Biopsy of osteitis lesion with positive bacterial culture	14 (47)
> 1 osteitis lesion	11 (37)
Psoriasis	1 (3)
Pustulosis palmoplantaris	21 (70)
Acne	3 (10)
Arthritis/oligoarthritis	20 (67)

Data presented as *n *(%) unless stated otherwise.

**Figure 1:**
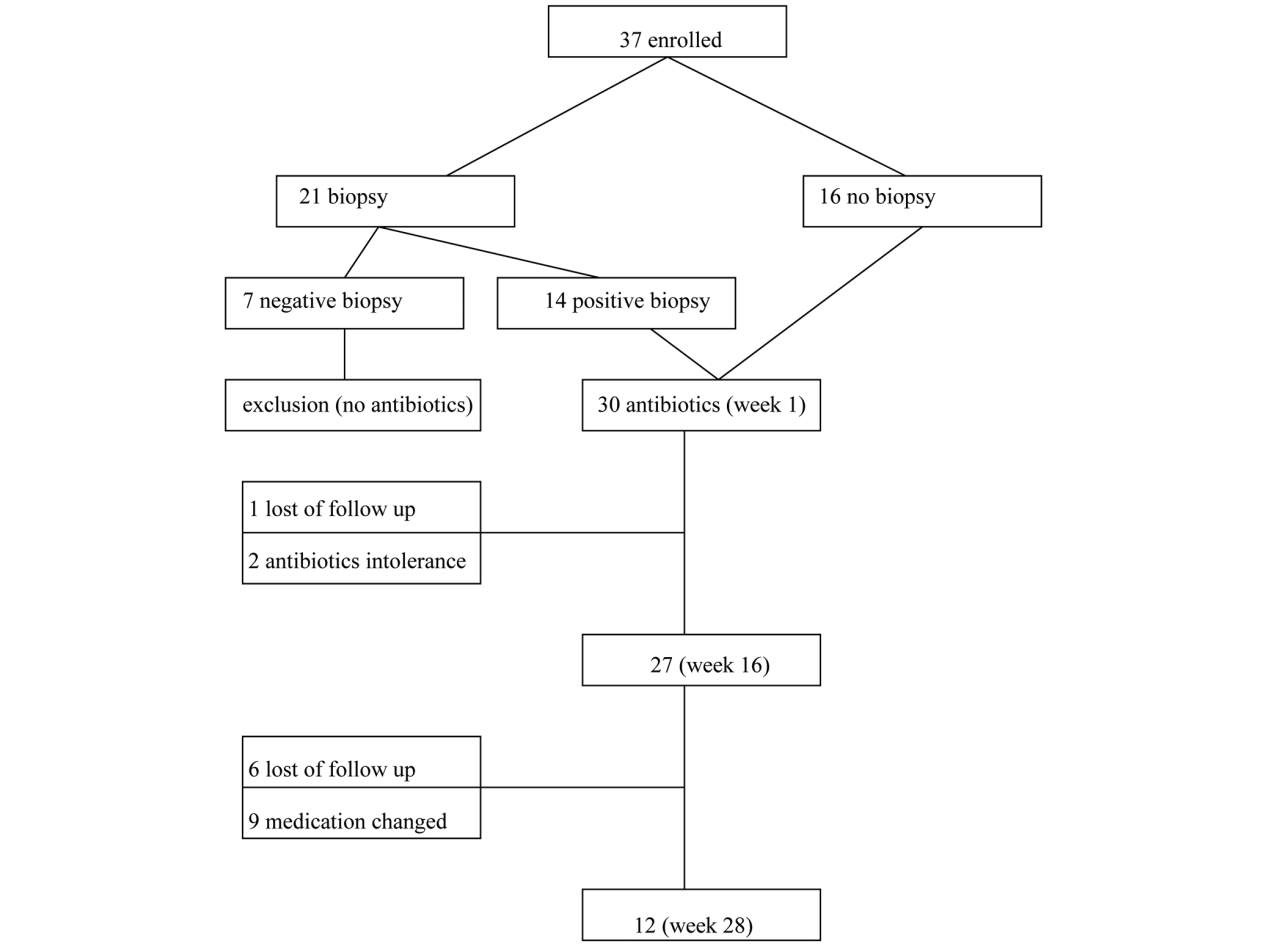
Flow chart from initial study enrollment to study follow-up for the treatment of SAPHO syndrome.

We enrolled 37 patients with SAPHO syndrome showing disease activity as determined by a scoring system based on the erythrocyte sedimentation rate (ESR), MRI findings, skin activity and osteitis activity (see below). All patients except those with a negative bacteriological finding (n = 7) in the needle biopsy of the osteitis lesion were offered treatment with azithromycin (alternatively with doxycycline or clindamycin). Thirty patients agreed to antibiotic treatment (Figure 1). Table 1 presents the demographic and clinical - pathological patient characteristics of these treated patients.

### Treatment regime

Twenty-two out of 30 patients were on antirheumatic medications without antibiotics, with a stable dosage of the respective drugs at least 4 weeks prior to the beginning of this study (outlined in Table 2). The 27 patients treated with azithromycin received a loading dose of 500 mg on six successive days, followed by 500 mg twice a week. After needle biopsy of the osteitis lesion, three patients from the antibiotics group showed a positive bacteriological culture for *P. acnes *resistant to azithromycin but sensitive to doxycycline in two patients and sensitive to clindamycin in one patient. Two patients therefore received doxycyline (100 mg daily) and one patient received clindamycin (300 mg daily) as the first-line antibiotic drug. Eight patients were without concomitant antirheumatic medication.

**Table 2:** Antirheumatic treatment of SAPHO patients on antibiotics at baseline

Medication except antibiotics	SAPHO patients (n = 30)
No medication	8 (27)
Nonsteroidal anti-inflammatory drugs^a^	10 (33)
Prednisolone	2 (7)
Dosage (mg/day)	5.0 (4.0 to 6.0)
Sulfasalazine^b^	1 (3)
Methotrexate	8 (27)
Dosage (mg/week)	12.8 (10.0 to 15)
Etanercept^c^	3 (10)
Bisphosphonate^d^	3 (10)

Data presented as *n *(%) or mean (range). ^a^Nonsteroidal anti-inflammatory drugs in a stable daily dosage: diclofenac, 100 or 150 mg daily; naproxen, 1,000 or 1,500 mg daily; ibuprofen, 800, 1,200 or 1,600 mg daily; celecoxib, 200 mg daily. ^b^Sulfasalzine in a dosage of 2 g daily. ^c^Etanercept in a dosage of 50 mg weekly. ^d^Alendronat in a weekly dosage of 70 mg.

Twenty-seven patients completed the 16-week treatment with antibiotics, and one patient was lost at follow-up. Two patients dropped out because of intolerance to the antibiotic medication (one patient on azithromycin and one patient on doxycycline). After the end of the antibiotic therapy, only 12 patients completed the follow-up for a further 12 weeks, during which they were to continue the same antirheumatic medication - the study protocol did not allow antibiotics or any change of concomitant medication during this follow-up period. The remaining 15 out of 27 patients who had completed the antibiotic treatment according to the protocol were lost at follow-up (six patients) or changed medication (five patients who took additional nonsteroidal anti-inflammatory drugs daily, two patients who took additional corticosteroids orally, and two patients who had topic corticoid instillation of osteitis lesion).

### Definitions and assessment

In all patients the diagnosis of SAPHO syndrome was confirmed by the diagnostic procedure, including tests for C-reactive protein and the ESR, bone radioisotope scanning with technetium in two phases and MRI of osteitis lesions. The scoring system was assessed within 1 week prior to the initiation of the antibiotic therapy, after week 16 and in week 28 (or 12 weeks after ending antibiotic treatment), and included an elevated sedimentation rate (ESR), MRI of osteitis lesions, clinical activity of skin lesions and clinical activity of osteitis lesions as well as a Health Assessment Score (HAS).

The MRI score for the osteitis lesions ranged from 0 to 2 and was assessed by a radiologist: score 0 was defined as no bone marrow edema, osteal erosions or synovitis (with or without joint effusion) imaged by T1-weighted and T2-weighted magnetic resonance technique; score 1 defined as one of theses pathological findings; and score 2 defined as more than one finding. In cases of more than one osteitis lesion, the lesion with the highest score was used as reference. The radiologist was blinded to the clinical scores under the treatment during the follow-up. Both the skin activity score and the osteitis score of the patients (ranging from 0 to 6) were assessed by the treating physician according to a questionnaire for the interview and by physical examination. The HAS was evaluated by the patient and described the subjective disease activity during the last 7 days before the assessment, using a scale ranging from 0 (no activity) to 6 (highest activity).

### Bacteriological findings

In all investigations the skin above the biopsy area was free of pustulotic changes. After thorough disinfection, a small surgical skin incision of approximately 3 mm was made under local anesthesia before the biopsy needle was advanced under computed tomography guidance. This procedure was supposed to minimise the possibility of specimen contamination by skin colonisation of *P. acnes *and other skin saprophytes. The computed tomography-guided needle biopsies of the osteitis lesion were inoculated for transport and were referred to the Department of Microbiology of Hannover Medical School for further work-up as previously described [27]. The biopsies were transferred directly after grinding plated on solid media (Schaedler's agar, chocolate agar; Oxoid Unipath, Wesel, Germany). Media were incubated for a minimum of 14 days at 35 ± 2°C for detection of aerobic, microaerophilic, and anaerobic microorganisms, including fastidious microorganisms. Their susceptibility to antibiotics was tested according to the National Committee for Clinical Laboratory Standard Protocol. Bone specimens were also sent to the Department of Pathology of Hannover Medical School for histopathological examination. The histopathological preparation in all specimens excluded malignant cell proliferation.

### Statistical analysis

The study had two major end points: the change of scores for the ESR, MRI, the HAS, skin activity and osteitis activity after 16 weeks of antibiotics; and the changing of scores after the end of the antibiotic treatment period. All data were analysed using the SPSS statistical package [28]. Quantitative variables are expressed as the mean ± standard error. The scoring variables outlined in the mean ± standard error were compared using pairwise testing for differences between the results for scores of weeks 1 and 16, of weeks 16 and 28, as well as of weeks 1 and 28. Pairwise-testing *P *< 0.05 was considered statistically significant.

## Results

### Baseline characteristics

The demographic and clinical - pathological characteristics of the 30 patients selected for antibiotic treatment are presented in Table 1.

### Outcome after treatment in week 16

Table 3 presents the results of the SAPHO disease activity in 27 patients before and after the treatment with antibiotics in week 1 and week 16. After week 16, the scores for MRI (1.5 to 1.1, *P *= 0.01), for skin activity (3.2 to 1.2, *P *= 0.01), for osteitis activity (4.0 to 2.1, *P *= 0.02), and the HAS (3.3 to 2.1, *P *= 0.01) decreased significantly in the antibiotics group.

**Table 3:** Activity scores of SAPHO patients in weeks 1 and 16 with antibiotic treatment

Activity score	Week 1	Week 16	*P *value (95% confidence interval)^a^
Skin	3.2 ± 0.4 (0 to 6)	1.2 ± 0.3 (0 to 4)	0.01 (1.29 to 2.64)
Osteitis	4.0 ± 0.3 (2 to 6)	2.1 ± 0.3 (0 to 6)	0.02 (1.25 to 2.40)
Erythrocyte sedimentation rate	24.1+-2.6 (9 to 45)	22.8+-3.1 (11 to 38)	0.34+-2.9 (- 0.20 to 0.53)
MRI	1.5 ± 0.1 (0 to 2)	1.1 ± 0.1 (0 to 2)	0.01 (0.24 to 0.88)
Health Assessment Score	3.3 ± 0.8 (0 to 6)	2.1 ± 0.4 (0 to 6)	0.01 (1.43 to 2.49)

Data presented as the mean ± standard error (range); n = 27. ^a^Pairwise test (value week 1 vs. value week 16).

### Outcome 12 weeks after the end of the antibiotic treatment

**Figure 2:**
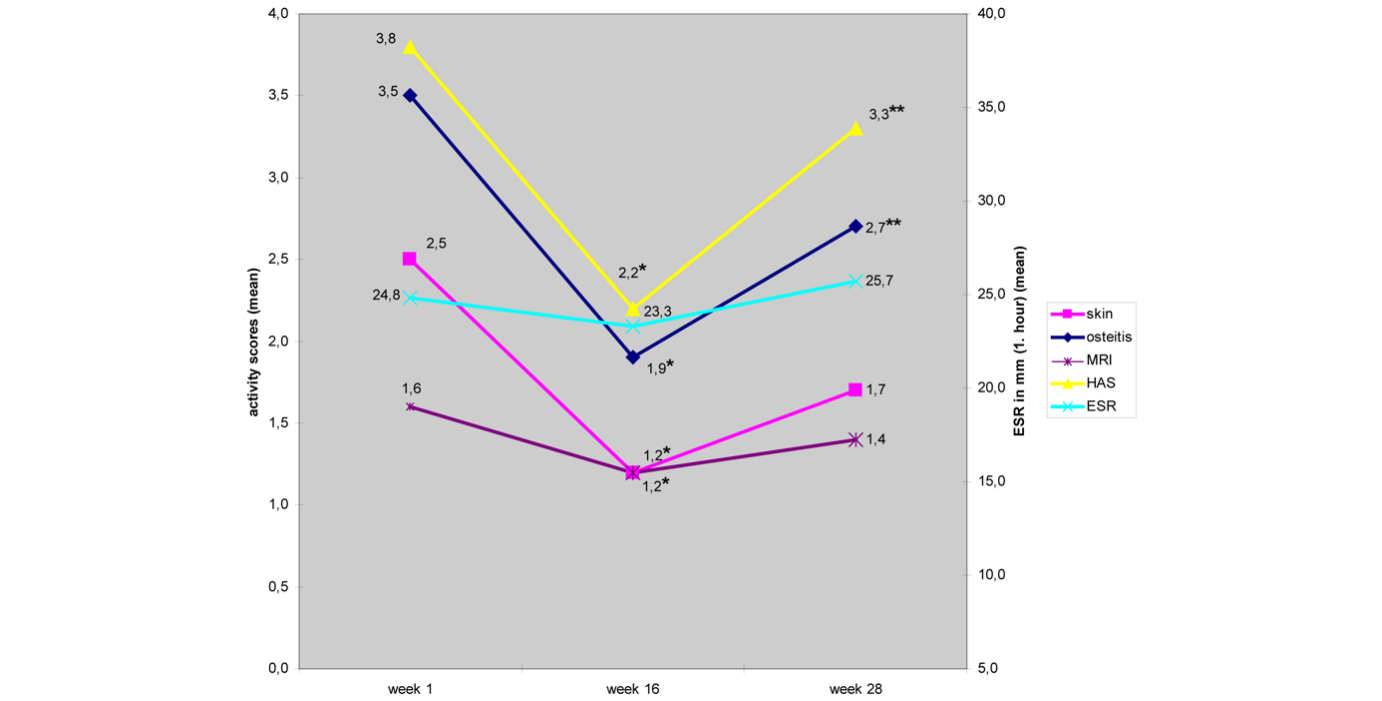
Activity scores and erythrocyte sedimentation rate values (mean) of 12 SAPHO patients treated with antibiotics. *differences of the values between week 1 and week 16: *P *< 0.05; **differences of the values between week 16 and week 28: *P *< 0.05; HAS = Health Assessment score; ESR = Erythrocyte sedimentation rate; MRI = Magnet resonance imaging.

Table 4 presents the outcome 12 weeks after the end of antibiotic treatment (week 28 after beginning of the study) of the 12 SAPHO patients who completed the follow-up for whom the activity scores were available at that time point. As shown in the table, compared with week 16, the follow-up scores in week 28 were increased with the respect to the MRI scores (1.2 to 1.4, *P *= 0.08), the skin activity scores (1.2 to 1.7, *P *= 0.11), the osteitis activity scores (1.9 to 2.7, *P *= 0.01), and the HAS (2.2 to 3.3, *P *= 0.02) from week 16 to week 28 (Figure 2). The comparison of the scores in week 1 and week 28 in these 12 patients, however, resulted in no significant differences: skin activity scores, 2.5 to 1.7 (*P *= 0.09); osteitis activity scores, 3.5 to 2.7 (*P *= 0.15); ESR, 24.8 to 25.7 (*P *= 0.34); MRI scores, 1.6 to 1.4 (*P *= 0.19); and HAS, 3.8 to 3.3 (*P *= 0.11).

**Table 4:** Activity scores in weeks 1, 16 and 28 for SAPHO patients treated with antibiotics

Activity score	Week 1	Week 16	Week 28	*P *value (95% confidence interval)
Skin	2.5 ± 0.5 (1 to 6)	1.2 ± 0.5 (0 to 4)	1.7 ± 0.5 (0 to 5)	0.01 (1.31 to 2.77)^a^
				0.11 (-1.43 to -0.24)^b^
				0.09 (-0.17 to 1.84)^c^
Osteitis	3.5 ± 0.4 (1 to 6)	1.9 ± 0.4 (0 to 4)	2.7 ± 0.5 (0 to 6)	0.01 (1.30 to 2.63)^a^
				0.01 (-1.43 to -0.24)^b^
				0.15 (-0.31 to 1.81)^c^
Erythrocyte sedimentation rate	24.8+-3.3 (9 to 45)	23.3+-2.8 (11 to 36)	25.7+-2.4 (12 to 40)	0.43 (-0.13 to 0.30)^a^
				0.35 (-0.33 to 0.13)^b^
				0.34 (-0.20 to 0.53)^c^
MRI	1.6 ± 0.1 (0 to 2)	1.2 ± 0.1 (0 to 2)	1.4 ± 0.2 (1 to 2)	0.01 (0.44 to 0.89)^a^
				0.08 (-0.54 to 0.04)^b^
				0.19 (-0.15 to 0.65)^c^
Health Assessment Score	3.8 ± 0.4 (2 to 6)	2.2 ± 0.3 (0 to 6)	3.3 ± 0.4 (1 to 6)	0.01 (1.51 to 2.79)^a^
				0.02 (2.02 to 0.32)^b^
				0.11 (-0.16 to 1.32)^c^

Treatment finishing in week 16. Data presented as the mean ± standard error (range); n = 12. ^a^Pairwise test (value week 1 vs. value week 16). ^b^Pairwise test (value week 16 vs. value week 28). ^c^Pairwise test (value week 1 vs. value week 28).

## Discussion

To the best of our knowledge, this is the first interventional study to evaluate the efficacy of long-term antibiotic treatment in patients with SAPHO syndrome. The goal of the present study was to determine the therapeutic effect of antibiotic treatment over a period of 4 months. Our results show an effect of a 4-month treatment with azithromycin (also with doxycycline and clindamycin in one patient each) with respect to MRI findings and to the activity of skin disease and osteitis. Three months after the end of antibiotic treatment, however, these effects had disappeared. The observed changes in the SAPHO activity scores were so closely associated with the antibiotic treatment that a placebo effect is rather unlikely. Studies that include a placebo control group, however, are required in order to clarify the efficacy of antibiotic therapy of SAPHO syndrome.

Azithromycin was chosen as the first-line therapy because of a broad spectrum of antimicrobial activity that has been shown *in vitro *to be highly concentrated in various phagocytic cells and to be active against bacteria within these cells [29]. The uptake and magnitude of concentrations of the antibiotic in phagocytes over extended periods of time were therefore supposed to be interesting, particularly for the treatment of osteitis. Furthermore, the accumulation of azithromycin in phagocytic cells is suggested to contribute to a more effective eradication of phagocytised bacterial organisms [30]. Moreover, macrolids are known to have a wide range of anti-inflammatory mechanisms besides their antibiotic properties [31,32]. In line with these results, previous studies demonstrated that clinical isolates of *P. acnes *were highly susceptible to azithromycin. To what extent the change of SAPHO activity observed in our study was due to a specific antibiotic effect or a more antiphlogistic effect of the antibiotics, or both, cannot be derived from our data [33].

There are only few studies investigating the effect of antibiotic treatment in a small number of SAPHO patients. Bellara and colleagues described two patients with SAPHO syndrome with a positive response to long-term doxycylin treatment [34]. Schaeverbeke and colleagues reported one case of successful treatment of a SAPHO patient with azithromycin [35]. In addition, a successful treatment regime of SAHPO syndrome with sulfamethoxazole/trimetoprim was previously described [7]. Kirchhoff and colleagues presented data for seven patients being treated successfully with azithromycin over 5 months [27]. A successful antibiotic treatment with azithromycin was also described for patients with CRMO [24]. For the time being, these case reports and uncontrolled observations are consistent with our results.

The treatment regimes of the present study included in most of the patients a combination therapy of conventional antirheumatic drugs and antibiotics. Based on the specific pharmacogenetic characteristics of azitromycin, sulfasalazine, and methotrexate, potential drug interaction was expected. No specific side effects of these drug combinations were detected, however, in the present study population of SAPHO patients. Whether the efficacy of antibiotics was possibly altered (weakened or increased) cannot be derived from our data.

Whereas the majority of papers concerning antibiotic therapy in infectious diseases report a clear association between disease duration and success of antibiotic treatment, the data for this study population cannot determine such effects because of the heterogeneity of the disease duration, ranging from 1 to 27 years.

One important result of our present study data is the disappearance of the antibiotic treatment effect after discontinuation of these drugs. Osteitis activity and the HAS increased statistically significantly, while skin activity and the MRI findings showed a strong trend towards increasing values. The comparison of the scores in week 28 with week 1 shows no statistically significant differences, indicating a return to baseline values after the end of antibiotic treatment. SAPHO syndrome, however, can be considered a basically relapsing - remitting disease. For this reason the data presented here cannot definitely exclude that a deterioration of the disease activity after antibiotic discontinuation could also be associated with a relapse of disease regardless of the antibiotic therapy. Nevertheless, the correlation between the ending of antibiotic therapy and increasing disease activity appears to be plausible. This correlation is further supported by the fact that 9 out of 27 patients were excluded from the study because of a change in antirheumatic medication between week 16 and week 28. In these patients an intensification of antirheumatic therapy was required to treat the deterioration of SAPHO syndrome activity. Combined, these data place SAPHO syndrome in a range of chronic inflammatory arthropathies with a potential need for permanent therapy, and raise the question of whether there is a need for discussion of how to prevent microbial resistance and disease escape by periodical drug administration or discontinuation.

## Conclusions

For the period of application the antibiotic therapy appears to control the disease activity of SAPHO syndrome, whereas discontinuation of antibiotic therapy seems to be associated with disease deterioration. In what way a permanent administration of antibiotic therapy (beyond 4 months) is a promising treatment option for SAPHO syndrome cannot be derived from our data.

## Abbreviations

CRMO: chronic recurrent multiple osteomyelitis; ESR: erythrocyte sedimentation rate; HAS: Health Assessment Score; MRI: magnetic resonance imaging; SAPHO: synovitis, acne, pustulosis, hyperostosis, and osteitis; TNF: tumor necrosis factor.

## Competing interests

The authors declare that they have no competing interests.

## Authors' contributions

GA, ADW and HZ conceived and designed the study. GA, JV, ADW and OK contributed to the acquisition of the samples or study data by patient interview or chart reviews. GA, JV, ADW, OK and MP were involved in the interpretation of the clinical data. TK and HR were responsible for the data interpretation of the radiological findings. All authors read and approved the final manuscript.
